# Surgically-Induced Necrotizing Scleritis After Pars Plana Vitrectomy: A Case Report

**DOI:** 10.7759/cureus.58652

**Published:** 2024-04-20

**Authors:** Shafiq Tanveer, Vemparala Priyatha, Asna Tahir, Devina Ramesh, Moram Mahmoud, Safina Tanveer

**Affiliations:** 1 Ophthalmology, Khyber Medical College/Khyber Teaching Hospital, Peshawar, PAK; 2 Internal Medicine, All India Institute of Medical Sciences, Bhubaneswar, Bhubaneswar, IND; 3 Ophthalmology, Khyber Teaching Hospital MTI, Peshawar, PAK; 4 Medicine, Queen's University, Kingston, CAN; 5 Public Health, University of Medical Science and Technology, Khartoum, SDN; 6 Medical School, Hayatt University College, Khartoum, SDN; 7 Surgery, Khyber Medical College/Khyber Teaching Hospital, Peshawar, PAK

**Keywords:** delayed hypersensitivity, diabetes mellitus, infectious diseases, pars plana vitrectomy, ophthalmology, surgically-induced necrotising scleritis

## Abstract

Surgically induced necrotizing scleritis (SINS) is a rare delayed hypersensitivity reaction following ocular surgeries, characterized by pain and redness at the surgical site. While commonly reported in various ocular surgeries, its occurrence after vitreoretinal procedures remains infrequent. We present a case of a 61-year-old diabetic male who developed progressive scleral melting and uveal exposure two months after an uneventful 23-gauge vitrectomy for retinal detachment. The infectious and immunologic profile was negative. Despite aggressive medical and surgical interventions, the patient exhibited advancing scleral melting. The diagnostic challenge lies in determining the relative contributions of trauma, epithelial breakdown, immune activation, and infection in these patients. Our patient's uncontrolled diabetes potentially aggravated vascular disruption, contributing to delayed wound healing and immune complex deposition. The treatment involved topical steroids with broad-spectrum antibiotics, followed by conjunctival flap and oral corticosteroids. This case underscores the importance of early diagnosis, cautious immunosuppression, and thorough infection evaluation in managing postoperative scleritis. The limitations include a single culture test and the patient being lost to follow-up.

## Introduction

Surgically induced necrotizing scleritis (SINS), a delayed hypersensitivity reaction following ocular surgeries with scleral incisions, manifests as pain and redness at the surgical site, with onset ranging from days to years [1]. Although commonly associated with various ocular surgeries, such as cataract surgery, trabeculectomy, squint surgery, and scleral buckling, SINS is infrequent after vitreoretinal procedures. SINS occurrence following uncomplicated, 20-gauge three-port pars plana vitrectomy (PPV) without buckling is underreported, emphasizing the need for prompt and aggressive immunosuppression [2]. Here, we present a challenging case involving a patient with progressive scleral melting and exposure of the underlying uvea despite rigorous medical and surgical interventions. The individual had a history of multiple surgeries and uncontrolled diabetes mellitus, both recognized risk factors predisposing to recurrent injury with delayed wound healing, potentially triggering an aggressive SINS hypersensitivity reaction. This case underscores the complexities of SINS management and highlights the importance of early intervention, particularly in high-risk scenarios. The challenges lie in navigating the complex interplay of surgical factors and patient-specific conditions, contributing to the development of this rare and severe complication.

## Case presentation

A 61-year-old male with diabetes presented with sudden pain, redness, and decreased vision in his left eye (OS) two months post-retinal detachment surgery. The surgery involved a 23-gauge three-port pars plana vitrectomy (PPV), membrane peeling, endo laser, and silicone oil tamponade. His past ocular history included bilateral extracapsular cataract extraction performed 4 and 5 years ago in right eye (OD) and OS respectively, and two doses of anti-VEGF injections in the left eye at the site of pars plana 3.5 away from the limbus supero-temporally. Silicone oil removal from the site of pars plana had occurred 10 days before presentation, with no post-operative complications. His medical history included uncontrolled diabetes, neuropathic and nephropathic complications, and a below-the-knee amputation for a diabetic foot ulcer.

**Figure 1:**
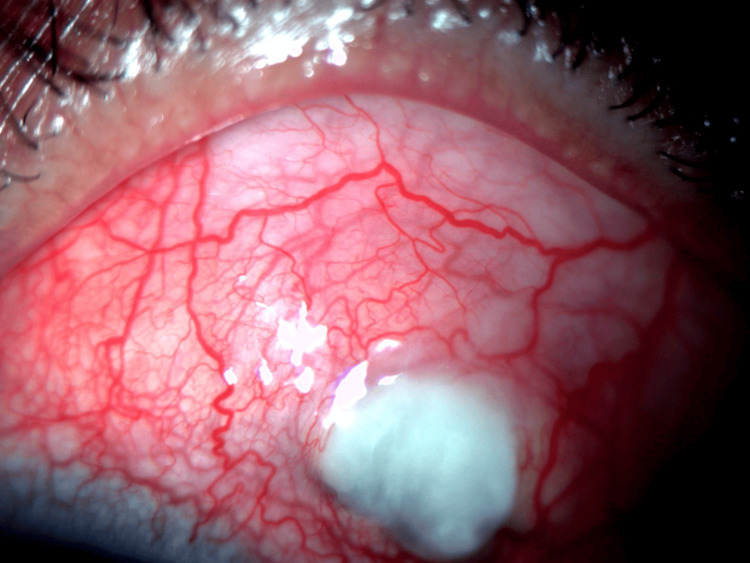
Coloured anterior segment photograph showed conjunctival hyperemia with circumcorneal congestion and generalized episcleral and scleral congestion with tortuous vasculature, scleral melting, and exposed uvea under melted sclera supero-temporally at the site of scleral port.

On examination, the best corrected visual acuity (BCVA) was 20/40 near vision of N6, and 20/200 near vision of N36 in the right and left eyes, respectively. The right eye showed reactive pupils with a hard posterior chamber intraocular lens in the sulcus. The anterior segment was deep and quiet. OS exhibited generalized conjunctival hyperaemia with circumcorneal congestion along with episcleral and scleral congestion with tortuous vasculature, scleral melting at the port site of PPV performed, and exposed uvea under melted sclera supero-temporally at the site of scleral port 3.5 mm away from the limbus at the pars plana (Figure 1).

The intraocular pressure (IOP) was 12 and 18 mmHg by Goldman tonometry in OD and OS. The anterior segment was quiet, with a clear cornea in both eyes (OU). The patient was pseudophakic (PPK) in both eyes. Fundus examination revealed an attached retina with laser marks in the OS. B-scan ultrasonography was normal in both eyes. A conjunctival swab along with a biopsy of the conjunctiva was performed prior to initiation of oral non-steroidal anti-inflammatory drugs (NSAIDs), topical moxifloxacin, topical corticosteroids, and cycloplegics. He was taking regular insulin and oral hypoglycemics for diabetes management.

**Figure 2:**
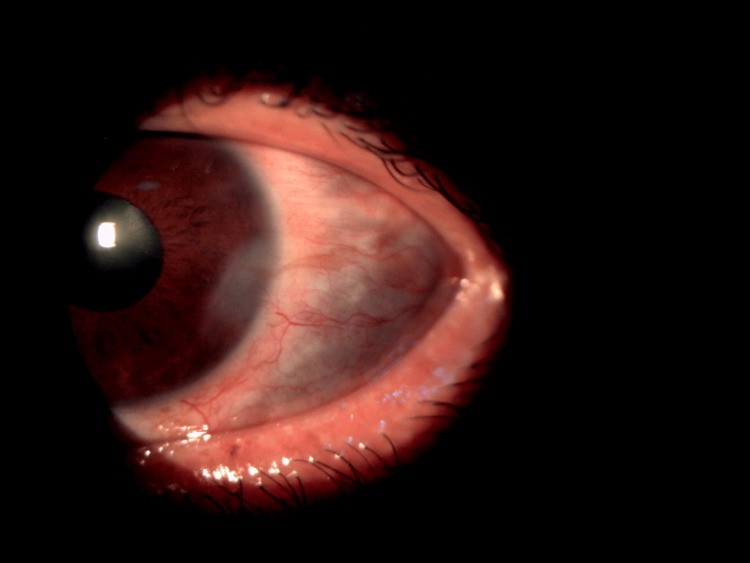
Coloured photograph of anterior segment showing multiple advancing areas of scleral thinning with exposed uveal tissue at the 1-week follow-up despite initiation of aggressive medical therapy

The conjunctival biopsy revealed no growth of any organisms after 72 hours on culture (chocolate, blood, and Sabouraud dextrose agar). Serum rheumatoid arthritis (RA) factor, antinuclear antibody (ANA), anti-neutrophil cytoplasmic antibodies (c-ANCA, p-ANCA), venereal disease research laboratory (VDRL), and treponema pallidum hemagglutination (TPHA) were negative. The erythrocyte sedimentation rate was 70 mm at the end of 1st hour. The Monteux test was negative as well. Laboratory values indicated uncontrolled diabetes with suboptimal HbA1c (11.01%), elevated urea (84 mg/dL), serum creatinine (1.4 mg/dL), and a decreased left ventricular ejection fraction (LVEF) of 28%. A multidisciplinary approach was undertaken for his uncontrolled diabetes, deteriorated LVEF, and deranged renal function with the Endocrinology, Nephrology, and Cardiology departments. At the one-week follow-up, the patient had improved symptomatically but on examination, the sclera showed multiple advancing areas of scleral thinning with exposed uveal tissue (Figure 2). A conjunctival flap was applied to the areas of scleral thinning.

The patient was diagnosed with surgically induced necrotizing scleritis (SINS) as the patient had undergone multiple ocular surgeries, didn't respond to the treatment, rapidly progressive scleral melting and thinning despite medical treatment, and had uncontrolled diabetes. Oral prednisolone (1 mg/Kg) was initiated, with a plan for dose reduction at one month. Unfortunately, the patient was lost to follow-up.

## Discussion

Surgically induced necrotizing scleritis (SINS) is an infrequent complication following vitreoretinal procedures, with a total of eight reported cases after such surgeries to date (as per a PubMed search) [1-9]. The potential pathogenesis of SINS involves organ-specific auto-reactivity causing capillary closure and tissue ischemia. This occurs in conjunction with surgery-induced scleral degradation and epithelial damage, observable under microscopic examination as granulomatous inflammation and fibrinoid necrosis [10]. Ocular trauma amplifies the lytic action of collagenases, resulting in collagen degradation, vascular disruption, local ischemia, and deposition of immune complexes. These events contribute to scleral necrosis, often compounded by delayed wound healing due to an abnormal immune response [11].

Diagnosing SINS is challenging due to the inherent limitations in the term, making it challenging to determine the relative contributions of trauma, epithelial breakdown, immune activation, and infection [12]. SINS commonly presents in eyes with a history of multiple surgeries. Donoghue et al. reviewed 52 eyes (43 patients) developing postoperative scleritis. It consistently emerged near surgical wounds, mostly following cataract extraction. The majority (75%) had multiple prior surgeries. The short latent period averaged 9 months, except for a subgroup with delayed onset after squint surgery. Scleritis was necrotizing in 96% of cases [13]. The primary therapeutic approach for SINS involves medical intervention with immunosuppressive agents such as oral corticosteroids, methotrexate, and cyclophosphamide [14]. Surgical management, including conjunctival flaps, as illustrated in this case, is also employed. The use of Tenon-conjunctiva flaps emerges as a highly effective strategy, promoting re-vascularization in the ischemic region and preventing further necrosis. Additionally, alternative options like corneal and scleral patch grafts, an amniotic membrane, and a collagen matrix are recognized as viable alternatives [7].

Das et al. reported a series of four SINS cases, where underlying comorbidities such as hypertension and diabetes were present. These conditions, known to potentially contribute to localized ischemia at the surgical wound site [5], were observed in the series, mirroring the situation in the patient discussed in this case. Uncontrolled diabetes in this case might have exacerbated vascular disruption, local ischemia, immune complex deposition, and delayed wound healing.

## Conclusions

In conclusion, maintaining a heightened suspicion for SINS in postoperative cases is vital for early diagnosis and effective treatment, preventing complications like staphyloma and scleral melting. Persistent or recurrent postoperative pain, unresponsive to standard anti-inflammatory measures, should raise suspicion of scleritis. While immunosuppression is crucial for managing postoperative necrosis, especially in cases without systemic immune disorders, a thorough investigation to rule out infection before initiating long-term immunosuppressive therapy is imperative. The study's limitations include a single culture test, potentially insufficient to completely exclude infection, and the inability to conduct extended follow-up due to the patient being lost to follow-up.
